# From Broken Models to Treatment Selection: Active Inference as a Tool to Guide Clinical Research and Practice

**DOI:** 10.32872/cpe.9697

**Published:** 2022-06-30

**Authors:** Lukas Kirchner, Anna-Lena Eckert, Max Berg

**Affiliations:** 1 Department of Psychology, Clinical Psychology and Psychotherapy, Philipps-University of Marburg, Marburg, Germany; 2 Department of Psychology, Theoretical Cognitive Science, Philipps-University of Marburg, Marburg, Germany

Computational theories have fundamentally changed the scientific understanding of how the mind works for both healthy and pathological experiences and behaviours. In this context, the active inference framework has gained considerable attention within the scientific community (Heins et al., 2022; Smith et al., 2022). As a process theory, it integrates complex phenomena, such as perception, learning, and action under a unified theory of Bayesian inference (Da Costa et al., 2020; Friston et al., 2017). Active inference has proven useful in modelling data from heterogeneous fields ranging from cognitive neuroscience to biology and general psychology (e.g., Friston et al., 2016, 2017). Its computational tractability and biological plausibility have also made it increasingly relevant to clinical psychology in recent years (e.g., Smith, Badcock, et al., 2021).

In active inference and related, Bayesian neurocomputational theories, it is assumed that individuals do not have direct access to the circumstances in their surroundings. Instead, they have to infer the (probabilistic) properties of their environment through action and perception by integrating prior information about their environment with ambiguous sensory input in a rational (i.e., Bayes-optimal) manner (Friston et al., 2016; Hohwy et al., 2008). The resulting “internal model of the world” (i.e., the agent’s beliefs about how certain sensory information relates to environmental conditions) shapes future perception (Friston, 2010) and enables agents to leverage the past to predict the future in an ever-changing environment (Badcock et al., 2017). In accordance with this perspective, perception, action, and learning are all subject to inferential processes on different timescales (Smith et al., 2022). Individuals thus take an active role in constructing their experiences, which they can further alter by actively changing their environment.

Whereas accurate internal models provide good predictions about which action sequences lead to preferred sensory observations, distorted internal models can lead to aberrant experiences and behaviours that may hinder the organism from achieving its goals (Badcock et al., 2017; Schwartenbeck et al., 2015). There is evidence that the internal models of individuals with mental disorders show substantial deviations from each other and from the models of healthy individuals. For example, recent accounts of depression have conceptualised patients' tendency to reappraise or disregard positive information in terms of highly precise and hence tenacious negative prior beliefs (Kube et al., 2020). This computational perspective has inspired novel ideas for the treatment of depression (e.g., Chekroud, 2015). Similar reconceptualisations with relevance for psychological treatments have been suggested for numerous mental disorders and health conditions, including psychosis (Sterzer et al., 2018), persistent somatic symptoms (Paulus et al., 2019), and eating disorders (Barca & Pezzulo, 2020).

In this context, the active inference framework offers the opportunity to formalise deviations in a person’s internal models, thus enabling a detailed description and operationalisation of relevant experiential and behavioural distortions (e.g., Montague et al., 2012). From our point of view, this could improve clinical research, diagnostics, and the treatment of mental disorders in several ways. Clinical research may benefit from a finely grained formalisation of deviant experiences and behaviours within the active inference framework because it opens up a possibility for studying aetiological mechanisms from a computational perspective (Stephan, Binder, et al., 2016). Because of their generative structure, computational theories enable the derivation of well-operationalised hypotheses about pathological processes in mental disorders and the computer simulation of aberrant experience and behaviour. In comparison with empirical data, researchers could thus rigorously formalise, simulate, and compare different mechanistic models of patients’ experiential and behavioural symptoms. Moreover, the active inference perspective could inform the diagnostics of mental disorders (or rather the diagnostics of patients’ implicit belief systems) by providing practitioners with individual estimates of their patients’ internal model parameters in disorder-relevant situations (Stephan, Bach, et al., 2016). For example, using probabilistic gambling tasks that distinguish between goal-directed information seeking and random exploration behaviour could provide clinicians with individual parameter diagnostics regarding the relationship between information seeking, reward sensitivity, and psychopathology in substance abusers (Smith, Schwartenbeck, et al., 2021). Such applications could not only strengthen a more transdiagnostic perspective on mental disorders, but also have tangible implications for treatment development and treatment selection. If we assume that therapeutic interventions may have different effects on patients' model parameters, finely grained operationalisation in the context of active inference could contribute to tailored interventions that target specifically these parameters. From the practitioner's perspective, it would be particularly important to investigate which tasks are likely to diagnose internal models that inform treatment selection and guide psychotherapy.

The active inference approach affords an improved mechanistic understanding of pathological processes in mental disorders. As a unifying process theory of brain and mind function, it brings together perception, learning, action, and decision making under the umbrella of a Bayesian principle, which predestines it for clinical application. Because of its high degree of formalisation and its flexibility, we believe that the active inference approach is well suited to functionally link heterogeneous clinical phenomena to patients’ internal belief systems. This will enable researchers to better differentiate and operationalise underlying mechanisms and tailor the diagnosis, aetiology, and treatment of mental disorders.
